# The Attitude and Knowledge of General Practitioners and Nurses Towards Severe Mental Illness in the Primary Care Facilities of the Seychelles Islands

**DOI:** 10.1192/bjo.2025.10165

**Published:** 2025-06-20

**Authors:** Lisa Kissubi-Chang-Time, Athanasios Hassoulas, Anna-Lisa Labiche

**Affiliations:** 1 Cardiff University School of Medicine, Cardiff, United Kingdom; 2 Cardiff University School of Medicine, Cardiff, United Kingdom; 3 Health Care Agency, Ministry of Health, Mahe, Seychelles

## Abstract

**Aims:** People with severe mental illness have a reduced life expectancy of 15–20 years compared with the general population. The current literature shows this vulnerable population are 2 to 3 times increased risk of dying from cardiovascular disease, 2 to 6 times more likely to die from respiratory disease, and an increased risk of chronic viral infections such as HIV and hepatitis C. Patient, medication, and healthcare system factors influence the morbidity and mortality of people with severe mental illness. Stigma and discrimination by healthcare workers is a key contributing factor. We conducted this novel study in the Seychelles Islands with the aim of assessing the attitude and mental health knowledge of general practitioners and nurses towards severe mental illness in all 16 government primary healthcare facilities. We also aimed to explore the association of attitude and knowledge variables with sociodemographic characteristics and compare the attitude and knowledge between the two groups. We hypothesized that the greater the knowledge and understanding of severe mental illness the more positive and supportive the attitude would be.

**Methods:** A probability-stratified sampling technique was utilised to recruit 42 doctors and 97 nurses. The exposure variables were the sociodemographic characteristics. The outcome variables were attitude which was measured using the Mental Illness: Clinician’s Attitude Scale (MICA) and knowledge which was measured using the Mental Knowledge Schedule (MAKS). Chi-square test was used to examine the association between the sociodemographic characteristics with the attitude and knowledge variables. The threshold of significance was set at p<0.05.

**Results:** 24 doctors and 64 nurses participated in the study with a response rate of 57.1% and 66% respectively. 66.7% (n=16) of the doctors were expatriates and 93.8% (n=64) of nurses were Seychellois (n=64, 93.8%). 54.69% (n=35) of the nurses had high knowledge and 58% (n=14) of the doctors had positive attitude. Male practitioners were more inclined to have a better knowledge of mental health. Doctors with postgraduate qualification had more positive mental health attitude. No statistically significant association was found between attitude and mental health knowledge in the participants.

**Conclusion:** The study has shown that half of the primary health workers had inadequate mental health knowledge and half of them had negative mental health attitude. Primary health workers lack training in the area of mental health. The key intervention is training in mental health. Additionally, recommendation may be made to revise the orientation programme for doctors and nurses entering the healthcare system in Seychelles.

